# BIOLITMAP: a web-based geolocated, temporal and thematic visualization of the evolution of bioinformatics publications

**DOI:** 10.1093/bioinformatics/bty967

**Published:** 2018-12-05

**Authors:** Adrián Bazaga, Alfonso Valencia, María- JoséRementeria

**Affiliations:** 1 Barcelona Supercomputing Center (BSC); 2 ICREA, Pg. Lluís Companys 23, Barcelona, Spain

## Abstract

**Motivation:**

The fast growth of bioinformatics adds a significant difficulty to assess the contribution, geographical and thematic distribution of the research publications.

**Results:**

To help researchers, grant agencies and general public to assess the progress in bioinformatics, we have developed BIOLITMAP, a web-based geolocation system that allows an easy and sensible exploration of the publications by institution, year and topic.

**Availability and implementation:**

BIOLITMAP is available at http://socialanalytics.bsc.es/biolitmap and the sources have been deposited at https://github.com/inab/BIOLITMAP.

**Supplementary information:**

Supplementary data are available at *Bioinformatics* online.

## 1 Introduction

It is increasingly important to place value on the scientific contribution of different areas of science and technology. The fast development of bioinformatics and its intrinsically interdisciplinary nature makes it especially difficult to visualize how the contribution of different application areas and institutions evolves. A visualization is increasingly important to facilitate the analysis and planning by institutions, grant agencies and other social actors.

To facilitate the exploration of the publications in the different research fields of bioinformatics, we present BIOLITMAP, a web-based geolocated system designed to assist in the exploration of the temporal and thematic evolution of the publications. BIOLITMAP captures worldwide bioinformatics research published by institutions, allowing the exploration by year, journal and specific bioinformatics topic. With BIOLITMAP, we are providing a global overview of the evolution of the different research lines, institutions and countries in bioinformatics, a new feature that was unavailable until now. Moreover, it provides a visual and interactive way to navigate easily over the information for users to find scientific publications based on a period of time, journal, topic and institution, order them, as well as visualize the articles, print the search results and see the related statistics. With this tool, we are providing a new baseline to explore and obtain the scientific articles in a manner that was not possible with any other previous tool, opening the possibility for further analysis over it.

## 2 Materials and methods

### 2.1 Dataset

This system is based on the data obtained from the *Scopus* (https://www.scopus.com/) database, since it is accepted as a reliable source of citations (Falagas *et al.*, 2008). For this first version of the system, we have included articles published in five representative journals which publish large numbers of bioinformatics papers: Bioinformatics OUP, BMC Bioinformatics, BMC Genomics, PLoS Computational Biology and Nucleic Acids Research. *Scopus* has two different categories for documents: ‘Articles’ and ‘Reviews’. In our search, we have applied a filter to retain only the documents of the type ‘Article’, excluding the ‘Reviews’. Consequently, Application Notes in Oxford Bioinformatics are included, as well as the special issues of the journals. The current database covers the years 2005–2017, comprising a total of 46 552 articles.

### 2.2 Institution geolocation

The metadata provided by *Scopus* was used for the geolocation of the institution responsible for each article. We assign article location based on the address of the institution for the corresponding author, including all the affiliations of all the possible corresponding authors of each article. The addresses have been geolocated using the Google Maps Geolocation API.

### 2.3 Topic modeling

As a first step, the text of the collected articles was cleaned in three phases: deletion of extremely infrequent and very frequent terms by counting the number of occurrences of each word, transformation of all the words to lowercase and removal of verbs and adjectives, applying stemming and removing punctuation, invalid symbols and stop words.

Since the category of each article is not provided by *Scopus*, the area of each paper was assigned with a topic modeling approach (Griffiths and Steyvers, 2004) derived from the application of the Latent Dirichlet Allocation algorithm (LDA) to the complete set of abstracts (Blei *et al.*, 2003). Informally, LDA represents a set of text documents in terms of a mixture of topics that generate words with a particular probability, theoretically following a Dirichlet distribution.

The LDA model selection was performed using the perplexity value, or what is homologous, the inverse likelihood, which tries to evaluate the capacity of generalization of the model. We have executed 10 different LDA models using values of *k* ranging from 5 to 50, sequenced in intervals of 5. From this models, we were able to determine which value of *k* produced the minimum value of perplexity, or the equivalent, the maximum value of likelihood, given the set of data. After that, we have measured the perplexity for each model according to the number of topics *k* (Supplementary Fig. S1), finding *k *=* *15 to be a meaningful number of topics.

The topics obtained from the LDA model were associated with the EDAM ontology (Ison *et al.*, 2013), that provides operations, data types and identifiers in bioinformatics, as well as the topics we finally used to classify the articles in 13 areas: DNA, Functional genomics, Mapping, Molecular genetics, Pharmacogenomics, Phylogeny, Proteomics, RNA, Sequence analysis, Structure analysis, Tools, Transcriptomics and Molecular interactions, Pathways and Networks (find the distribution of the articles over the different journals in Supplementary Fig. S2 and the EDAM graph depicting the topics ontology in Supplementary Fig. S3). We matched the obtained topics from the LDA model to the EDAM ontology concepts by manual inspection of the significant terms (Supplementary Table S1). In some cases, we joined LDA topics in a single EDAM category, reducing them from 15 to 13 categories at the end. For instance, one of the LDA topics concerned with software tools was merged in the class of ‘Tools’. The assigned ontology categories were, also, informed by carrying a literature analysis using the keywords associated with each of the topics (Supplementary Table S2).

## 3 Exploring the map

**Fig. 1.**
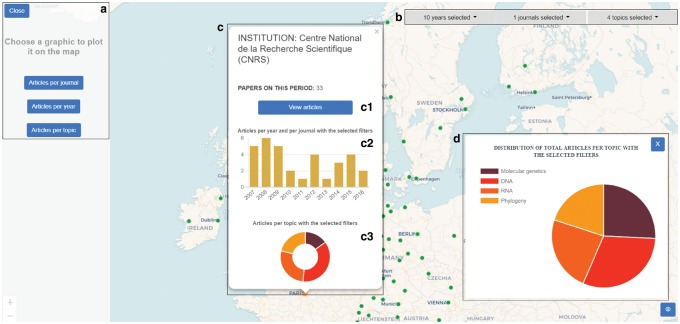
Snapshot of BIOLITMAP web-based application, showing some aspects of the application, namely: (**a**) it shows the available graphic options for the totals, (**b**) a widget intended to provide the user the ability to choose the filters of his/her interest: range of years, journals and bioinformatics topics, (**c**) the information window for each institution, opened when clicking on one of them and (**d**) an example global statistics plot showing the distribution of the publications in the selected five topics

A snapshot of the visualization is shown in (Fig. 1). Using the upper right widgets (Fig. 1b), the user can choose from the following: the period of years to visualize, specific journals of reference and the desired topics. The left side graphic options (Fig. 1a) allows the user to visualize the overall statistics (Fig. 1d).

In the map, the color of the institutions is proportional to the total number of publications for the selected option: green color for the range of 1 to 20 papers, orange for 21 to 50 and red for more than 50 papers. Furthermore, by clicking each institution in the map, the number of papers in the selected period for the corresponding institution are shown, together with an option (Fig. 1c1) to list and print the articles (example in Supplementary Fig. S4), the number of articles per year and per journal published by the institution (Fig. 1c2) and the number of articles per topic (Fig. 1c3). The user should notice that the filters (Fig. 1b) are applied to the data feeding the map, which affects both, the overall statistics and graphics (Fig. 1a and d) and the per-institution statistics and graphics (Fig. 1c). 

As a use case, a user might want to see the publications in a specific journal, as for instance Bioinformatics OUP, in the period 2007–2016, in the topics of Molecular genetics, DNA, RNA and Phylogeny. To do so, the user would just need to select the desired filters, wait for the system to show the results and click, for instance, the ‘Articles per topic’ graphic option. The graphic will show the number of papers per topic with the selected filters, which are 313 corresponding to Phylogeny, 372 to Molecular genetics, 483 to DNA and 333 to RNA. The user might also want to use the rest of the graphic options: ‘Articles per journal’ or ‘Articles per year’, as well as focusing the attention to a particular institution. For the latter case, the user just needs to navigate to the institution in the map and click it, an action that will display the related information for the clicked institution. In this example, CNRS has produced 33 papers, with an equal distribution in the 4 selected topics.

### 3.1 Limitations and future work

The main limitations are related with the correctness of the automatic classification of papers within one of the subject areas, and the possible inclusion of papers that do not belong to bioinformatics properly speaking. Moreover, the attribution to specific institutions is not free of ambiguities, such as the distortion caused by large umbrella institutions, e.g. CNRS, in the example. This difficulty that can be extended to the selection of the authors and institutions to which to assign the publications, is always a controversial topic.

We plan to extend the spectrum of analyzed journals and include additional filters to select bioinformatics papers. Finally, given the recent interest in mapping and analyzing the contribution of bioinformatics tools, we will explore the application of the system to the visualization of the evolution of the bioinformatics tools described in the publications.

## Funding

This work was supported by grants INB (PT17/0009/0001–ISCIII-SGEFI/ERDF), ELIXIR-EXCELERATE, funded by the European Commission within the Research Infrastructures programme of Horizon 2020, grant agreement number 676559 and the joint BSC-IRB-CRG Program in Computational Biology and the Severo Ochoa Award (SEV 2015-0493).

*Conflict of Interest*: One of the authors (Alfonso Valencia) is editor in Oxford Bioinformatics. Alfonso recommends Janet Kelso as assigned editor.

## Supplementary Material

bty967_Supplementary_Data

## References

[bty967-B1] Blei D.M. et al (2003) Latent Dirichlet allocation. J. Machine Learn. Res., 3, 993–1022.

[bty967-B2] Falagas M.E. et al (2008) Comparison of PubMed, scopus, web of science, and google scholar: strengths and weaknesses. FASEB J., 22, 338–342. 17884971 10.1096/fj.07-9492LSF

[bty967-B3] Griffiths T.L., Steyvers M. (2004) Finding scientific topics. Proc. Natl. Acad. Sci., 101 (Suppl. 1), 5228–5235. 14872004 10.1073/pnas.0307752101 PMC387300

[bty967-B4] Ison J. et al (2013) EDAM: an ontology of bioinformatics operations, types of data and identifiers, topics and formats. Bioinformatics, 29, 1325–1332. 23479348 10.1093/bioinformatics/btt113 PMC3654706

