# Supplementary material for: BIOLITMAP: a web-based geolocated, temporal and thematic visualization of the evolution of bioinformatics publications
Source: Bioinformatics. 2018 Dec 5;35(14):2518–20. doi: 10.1093/bioinformatics/bty967 (PMC6612868; doi:10.1093/bioinformatics/bty967)
Supplement: bty967_Supplementary_Data [file bty967_supplementary_data.pdf]

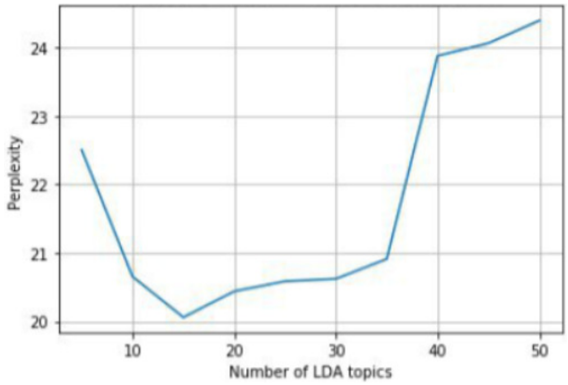

Figure S1: Perplexity for each LDA model according to the number of topics 'k', ranging from 5 to 50, sequenced in intervals of 5.

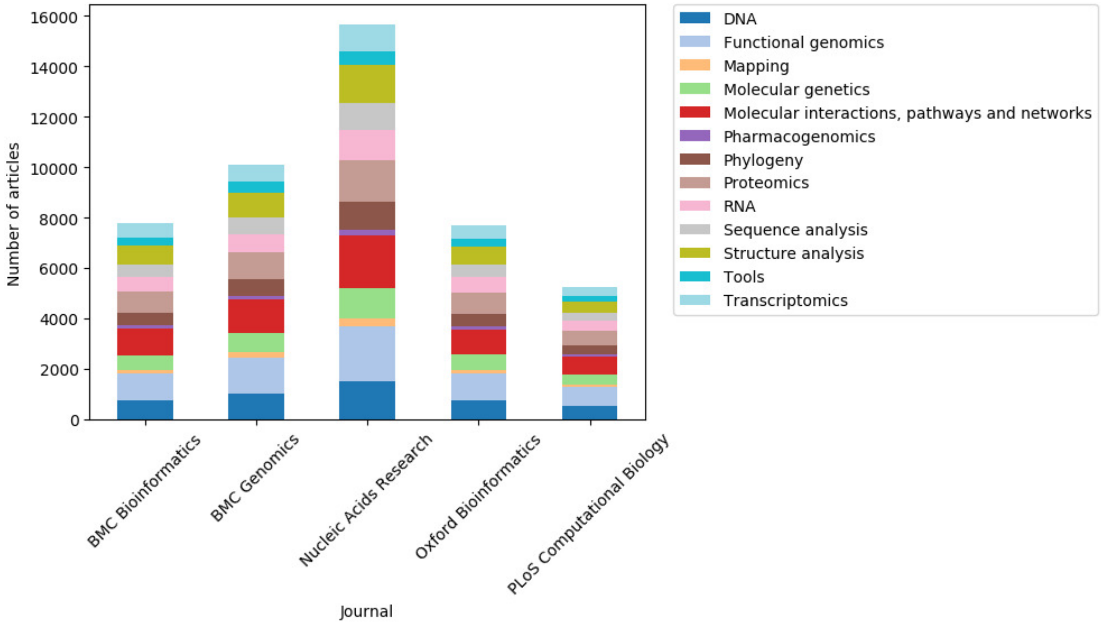

Figure S2: Distribution of the number of studied articles and their topics over the different journals.

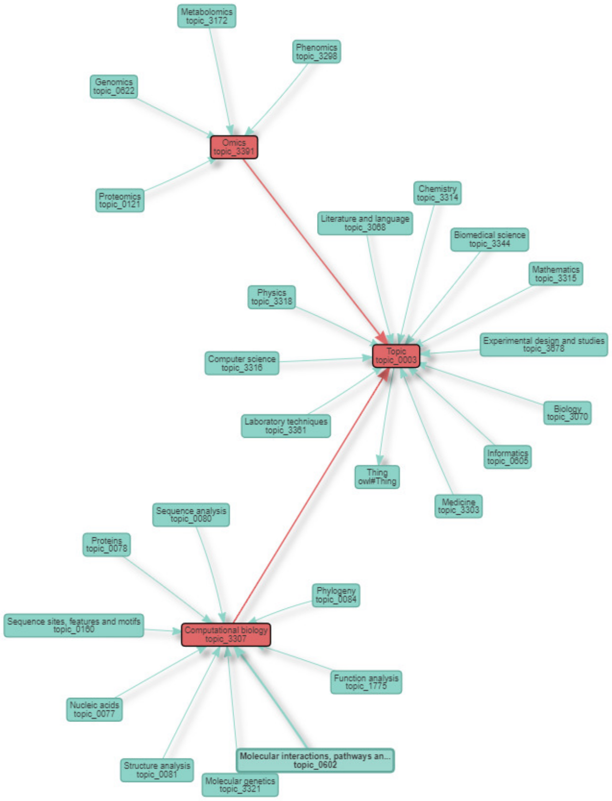

Figure S3: EDAM graph representation of the omics and computational biology topics.

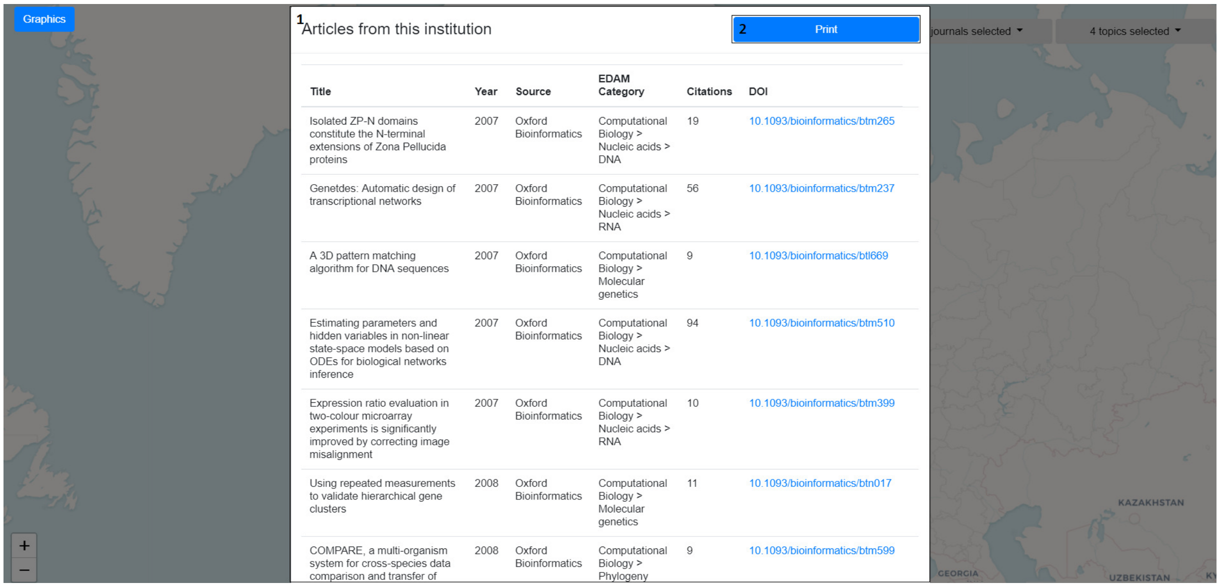

Figure S4: Listing of articles for a certain institution (in this example, CNRS) that appears when pressing the corresponding 'View articles' option. The following information for each of the articles, according to the selected filters, is displayed: title of the article, the year when the article was published, the source of the article (e.g. the journal name), the EDAM category we have assigned to it, the number of citations and the DOI of the article. Using the option 'Print' (2), the list will be converted into PDF format and downloaded, thus facilitating a way to download and share the information available on BIOLITMAP.

Table S1: Top 10 terms of the topics obtained by the LDA model and the assigned EDAM ontology topic for each one

Topic	Top 10 terms within the topic (in descending order of relevance)	
Topic 1	genome, region, property, element, analysis, finding, function, sequence, mechanism, specie	Omics > Genomics > Functional genomics
Topic 2	sequence, databases, datasets, analysis, element, finding, region, gene, function, property	Computational biology > Sequence analysis
Topic 3	structure, protein, function, analysis, finding, property, database, sequence, element, group	Computational biology > Structure analysis
Topic 4	condition, dynamic, group, mechanism, gene, finding, property, function, analysis, experiment	Computational biology > Molecular interactions, pathways and networks
Topic 5	pattern, finding, property, function, analysis, mechanism, region, group, element, dna	Computational biology > Sequence analysis > Mapping
Topic 6	cell, element, mechanism, finding, function, property, dna, analysis, region, condition	Computational biology > Molecular genetics
Topic 7	network, database, organism, property, gene, function, finding, analysis, protein, datasets	Computational biology > Molecular interactions, pathways and networks
Topic 8	protein, datasets, function, property, finding, gene, analysis, mechanism, group, disease	Omics > Genomics > Proteomics
Topic 9	rna, transcript, function, finding, property, mechanism, element, analysis, sequence, protein	Computational biology > Nucleic acids > RNA
Topic 10	specie, sample, analysis, gene, finding, group, mechanism, function, genome, database	Computational biology > Phylogeny
Topic 11	pathway, transcript, analysis, mechanism, gene, function, finding, database, condition, disease	Omics > Genomics > Transcriptomics
Topic 12	disease, mechanism, function, finding, dna, property, effect, analysis, condition, datasets	Omics > Genomics > Pharmacogenomics
Topic 13	experiment, effect, mutation, datasets, finding, analysis, mechanism, property, gene, function	Computational biology > Structure analysis
Topic 14	tool, datasets, analysis, database, function, gene, finding, group, sample, genome	Tools
Topic 15	dna, property, finding, protein, sequence, function, element, analysis, cell, condition	Computational biology > Nucleic acids > DNA

Table S2: Number of articles per topic over the period 2005-2017

Topic / Year	Functional genomics	Sequence analysis	Structure analysis	Molecular interactions, pathways and networks	Mapping	Molecular genetics	Proteomics	RNA	Phylogeny	Transcr.	Pharmac.	Tools	DNA
2005	287	122	174	275	40	156	225	150	129	134	38	71	217
2006	377	170	259	363	52	179	248	194	171	183	30	93	278
2007	397	204	314	385	70	232	315	228	197	199	59	119	283
2008	435	218	295	467	79	237	372	257	216	223	31	117	336
2009	527	252	390	524	89	279	400	272	290	257	55	156	384
2010	514	222	322	472	81	263	365	287	225	232	52	156	333
2011	513	261	362	470	78	282	393	293	224	265	54	152	361
2012	545	267	391	531	89	302	444	316	281	246	56	159	390
2013	545	253	402	529	87	322	423	271	266	282	45	143	397
2014	672	302	434	602	98	348	499	358	330	290	72	184	464
2015	606	316	422	644	99	353	499	356	314	345	67	191	439
2016	530	235	336	497	91	299	395	300	241	291	50	155	350
2017	451	201	285	451	69	274	370	283	235	244	54	127	341
